# Photoactivated TiO_2_ Nanocomposite Delays the Postharvest Ripening Phenomenon through Ethylene Metabolism and Related Physiological Changes in *Capsicum* Fruit

**DOI:** 10.3390/plants11040513

**Published:** 2022-02-14

**Authors:** Arijit Ghosh, Indraneel Saha, Masayuki Fujita, Subhas Chandra Debnath, Alok Kumar Hazra, Malay Kumar Adak, Mirza Hasanuzzaman

**Affiliations:** 1 Plant Physiology and Plant Molecular Biology Research Unit, Department of Botany, University of Kalyani, Kalyani, Nadia 741235, West Bengal, India; arijitgo625@gmail.com (A.G.); indraneelsaha92@gmail.com (I.S.); 2 Laboratory of Plant Stress Responses, Faculty of Agriculture, Kagawa University, Miki-cho, Kita-gun, Kagawa 761-0795, Japan; 3 Department of Chemistry, University of Kalyani, Kalyani, Nadia 741235, West Bengal, India; subhasd81@gmail.com; 4 IRDM Faculty Centre, Ramakrishna Mission Vivekananda Educational, Research Centre (RKMVERI), Ramakrishna Mission Ashrama, Narendrapur, Kolkata 700103, West Bengal, India; alokhazra8@gmail.com; 5 Department of Agronomy, Faculty of Agriculture, Sher-e-Bangla Agricultural University, Dhaka 1207, Bangladesh

**Keywords:** ethylene, elicitors, fruit ripening, ACC synthase/oxidase, GC-MS, polyamines

## Abstract

Capsicum is one of the most perishable fruit which undergo rapid loss of commercial value during postharvest storage. In this experiment our aim is to evaluate the effect of photoactivated TiO_2_ nano-particle complexed with chitosan or TiO_2_-nanocomposite (TiO_2_-NC) on extension self-life of *Capsicum* fruit and its effect on related morphological, physiological and molecular attributes at room temperature (25 °C). Initially, TiO_2_-NC coated fruits recorded superior maintenance of total soluble solids accumulation along with retention of firmness, cellular integrity, hydration, color etc. On the extended period of storage, fruit recorded a lower bioaccumulation of TiO_2_ in comparison to metallic silver over the control. On the level of gene expression for ethylene biosynthetic and signaling the TiO_2_-NC had more regulation, however, discretely to moderate the ripening. Thus, ACC synthase and oxidase recorded a significantly better downregulation as studied from fruit pulp under TiO_2_-NC than silver. On the signaling path, the transcripts for Ca*ETR1* and Ca*ETR2* were less abundant in fruit under both the treatment when studied against control for 7 d. The reactive oxygen species (ROS) was also correlated to retard the oxidative lysis of polyamine oxidation by diamine and polyamine oxidase activity. The gene expression for hydrolytic activity as non-specific esterase had corroborated the development of essential oil constituents with few of those recorded in significant abundance. Therefore, TiO_2_-NC would be reliable to induce those metabolites modulating ripening behavior in favor of delayed ripening. From gas chromatography-mass spectrometry (GC-MS) analysis profile of all tested essential oil constituents suggesting positive impact of TiO_2_-NC on shelf-life extension of *Capsicum* fruit. Our results indicated the potentiality of TiO_2_-NC in postharvest storage those may connect ethylene signaling and ROS metabolism in suppression of specific ripening attributes.

## 1. Introduction

Fruit ripening is a unique process in plant biology where differential gene expression leads to a set of cellular and biochemical events, mostly catabolic in nature. Those are regulated throughout the ripening period with some metabolic fluxes under coverage of growth regulators such as auxin, ethylene (ET), gibberellin, abscisic acid and kinetins [1]. Ethylene, the key hormone is responsible for respiratory burst accompanying other cellular changes hastening the ripening of fleshy fruit. The changes during ripening essentially include hydrolysis of storage polysaccharides, turnover of proteins, conversion of pigments, de-acidification into organic acids, hydrolysis of esters leading to softening of pulp tissues [2]. Regardless of climacteric and non-climacteric types ET evokes several signaling cascades for fruit senescence through gene expression. Mostly those govern catabolic processes of biomolecules which may expedite ripening within postharvest storage [3,4]. Therefore, ET-mediated cellular reactions have principally been in quest for identification of specific gene(s) to alter metabolic functions for enhanced shelf life [5]. Likewise, key biosynthetic genes, viz. ACC synthase and ACC oxidase are targeted by synthetic residues to inhibit their activity to reduce postharvest losses. The metallic silver (Ag) has been a choice for biosynthesis and functional inhibition for ET activities [6]. At the cellular level, ET mediated signaling in ripening fruit are also blocked the ET receptors: *ETR1*, *EIN3*, *CTR1*, etc. In downstream of ET signaling identification of specific transcription factors (*AP2/ERF*) to their corresponding *cis*-elements is another point of approach for delayed senescence/ripening under storage [7]. Ethylene regulated such genes are diverse, still, principally falls under wall hydrolyzing/depolymerization (softening of texture), sugar-acid interconversion (changes in taste), secondary metabolite turnover (for aroma) related genes. In the background of ET sensitivity, the climacteric and non-climacteric types are characteristically differed with the presence or absence of auto-catalytic ET generation [8]. Moreover, the regulation of ET also governs the changes of metabolic flux that correspondingly contribute to the sensory character of ripening. Despite this, in postharvest biology, exogenous application of ET might be aligned to several cellular responses for softening of non-climacteric fruit also [9]. The major focus has been paid on cell wall depolymerization, ET generation and pigment biosynthetic enzymes as responsive genes were identified from non-climacteric fruit such as pepper [10].

The commercial practices to retard the postharvest softening are materialized by regulation of the ripening specific pathways. Ethylene would be the key one in a system to avoid direct exposure to fruit or minimization ET induced ripening phenomenon in the tissues [11]. Many approaches are established to down regulate ripening of the fruit by synthetic inhibitors, action inhibitors of ET such as 1-amino vinyl glycine, Ag^+^ ion, respectively, are the most common. The other physical and chemical treatments through oxidation, decomposition and adsorption of gas are not bio-compatible for the corrosive nature of chemicals used [12,13]. The adsorption technologies with activated carbon in various forms as powder/granular, fibers, zeolite, silica gel, electron-deficient based compounds such as dienes and trienes are very time consuming as well as complicated device-based in application [14]. So, any system qualified for ET scavenging if proves non-toxic, less absorbing, biocompatible for fruit quality must be in pursuit for postharvest storage. In the modern state-of-art the exercise of nanocomposite with metallics is important to scavenge ET by complete oxidation [15]. In an application of TiO_2_-NP under UV radiation observed a significant removal of gas in in vitro system. This metal oxide produces superoxide and hydroxyl radicals and other reactive oxygen species (ROS) under UV excitation on its nano-surface. The produced ROS is more effective to oxidize ET into CO_2_, H_2_O, regardless of room/low temperature, high humidity and no other specific environments. It is also noteworthy that TiO_2_ within nano ranges varies ROS generation with surface bridging OH^•^. Moreover, there recorded an intrinsic correlation for ROS activation under UV illumination for TiO_2_ nanocomposite with oxidation of ET [16]. So, ROS generation and its involvement in the ripening process is expected to be modulated by TiO_2_ in nano forms.

Many investigations have been carried out on effect of bulk TiO_2_ as well as TiO_2_-NC on postharvest shelf life of fruit such as mango, tomato, cucumber etc. [17,18]. On the other hand, chitosan/TiO_2_-NP composite also can effectively protect grapes from fruit rotting micro-organism [19]. Although TiO_2_-NC have been used studies in various fruit and vegetable, there is almost no data regarding its effect on quality of *Capsicum* fruit during postharvest storage.

In this paper, we focused on effects of TiO_2_-NC coating on quality indices, physiochemical properties and molecular characteristics of *Capsicum* fruit during postharvest storage at 25 °C for 7 d. In this investigation, we have evaluated the effectivity of TiO_2_-NC for ROS generation, polyamine metabolism, wall loosening enzyme, volatile substances and gene expression for ET metabolism on delayed ripening during postharvest storage.

## 2. Materials and Methods

### 2.1. Plant Material and Treatments

Chilli or *Capsicum* fruits of Tejaswani cultivar were brought in mature green stage from a local agricultural farm, then checked for any deformity, infection, damage and disinfected with 1% sodium hypochlorite solution followed by a repeated wash with deionized water. Treatment sets for the fruits are as follows: control (treated with distilled water), treated with 2 mg mL^−1^ silver solution (silver), coated with 1% chitosan solution containing 10 g L^−1^ TiO_2_-nanoparticle or TiO_2_ nanocomposite (TiO_2_-NC). Samples were kept inside separate quartz bottles equipped with airtight cap containing rubber septa with 3 replicates per treatment and 30 fruits in each replicate. All the beakers are kept under UV-A (365 nm) led light for 7 d at 25 °C. Sampling was carried out after 7 d taking at least three replicates per treatment and stored in −80 °C for further analysis. All results are expressed on fresh weight (FW) basis.

### 2.2. Study of Fruit Surface Morphology through Scanning Electron Microscopy (SEM)

Fresh samples from each treatment were cut into manageable pieces followed by dehydration through critical point drying method with K850 (Quarum Technologies, Switzerland). After drying samples were placed on metallic stubs and coated with 30 nm thick gold with plasma sputter coater (MNT-JS1600, MNT, Canada). Finally, surface morphology of the coated samples was analyzed through SEM (GeminiSEM 450, Zeiss, Oberkochen, Germany) according to [20].

### 2.3. Quality Parameters

#### 2.3.1. Firmness of Fruit Coat

The firmness of fruit coat was measured by handheld penetrometer (GY-1, Wenzhou Tripod Instrument Manufacturing Ltd., China) with 8 mm wide stainless-steel plunger and measurements were carried out at 3 different points on fruit surface opposite to each other and the average value was expressed in N.

#### 2.3.2. Color of Fruit Coat

Fruit from each treatment 3 replicates were taken and the color of each fruit at 3 different points was measured by chroma meter (CR-400, Konica Minolta, Osaka, Japan). The color was reported as hue angle (°).

#### 2.3.3. Percentage of Water Loss

This was measured in gravimetric method comparing total water content (difference of weight of the fruit before and after drying in hot air oven at 50 °C for 48 h) and the difference in weight of fruit at initial day and after 7 d of treatment taking 3 replicates from each treatment according to Scalisi and O’Connell [21].

#### 2.3.4. Total Soluble Solid Content

Five g of fruit from each replicate was taken, and fruit juice was extracted from pericarp region by pressing. The total soluble solid content was measured using refractometer (RHB-32ATC MCP, Japan) by putting a few drops of fruit juice on the detection surface.

#### 2.3.5. Quantification of Ti in Fruit

One g of fruit pericarp was digested with 10 mL tri acid mixture (H_2_SO_4_:HNO_3_:HClO_4_ = 3:3:1) and cooled. After cooling the concentrated acid mixture was diluted in 90 mL of ddH_2_O and Ti content was measured with ICP-MS (Agilent 7800, Agilent Technologies Inc., Santa Clara, CA, USA) against known standard according to Ghosh et al. [6].

### 2.4. Ethylene Measurement

One mL of gas sample from each replicate were drawn using as GC syringe from the head space of each vail containing rubber septum and the ET was quantified with a flame ionization detector gas chromatograph (GC-8A, Shimadzu, Japan) equipped with an active aluminum column T.R. Ethylene content was expressed in nM kg^−1^ s^−1^.

### 2.5. Preparation of RNA

One g of fresh pericarp tissue of fruit was frozen with liquid nitrogen and frozen tissue was crushed to powder with mortar and pastel. RNA was isolated from about 50 mg frozen powdered tissue using trizol (Trizin, GCC Biotech, West Bengal, India) RNA extraction method according to the manufacturer’s protocol. For removal genomic DNA isolated RNA was digested with DNase I (Qiagen) according to manufacturer’s instructions. Quantification of RNA was carried out by nano-drop spectrophotometer (Thermo Fisher Scientific, Wilmington, NC, USA) and integrity RNA samples were analyzed by electrophoresis in 1.5% agarose gel.

### 2.6. Semi-Quantitative PCR

For semi-quantitative PCR analysis, total RNA samples were reverse transcribed into cDNA using cDNA synthesis kit (GCC Biotech, India) following the manufacturer’s protocol in triplicate. cDNA samples are amplified by step one PCR system using PCR master mix (GCC Biotech, India) with specific primers. Gene-specific primer for *ACCS*, *ACCO*, *ETR1*, *ETR2* and *GAPDH* used according to [22] where *GPDH* gene used as control. The temperature for the PCR reaction was: initial denaturation 95 °C for 5 min, 35 × (denaturation at 95 °C 30 s, annealing at 55 °C for 30 s and extension at 72 °C for 30 s), final extension at 72 °C for 5 min. 2 uL of PCR product from each sample was run in 2% agarose gel and photographed for using Gel Doc system. Densitometry scanning was carried out using GelAnalyzer 19.1 (www.gelanalyzer.com, accessed on 11 December 2022 ) software.

### 2.7. Quantification of ROS

#### 2.7.1. Quantification of H_2_O_2_

One g of the sample from each treatment were crushed 0.01 M phosphate buffer (pH 7.0). After that the homogenate is centrifuged at 12,000× *g* for 15 min and obtained supernatant was mixed with chromate reagent [19]. The mixture was then kept inside a boiling water bath until the development of greenish color and quantification of H_2_O_2_ was carried out spectrophotometrically at 570 nm according to Ghosh et al. [23]. The result was expressed as µmol g^−1^ FW.

#### 2.7.2. O_2_^•−^ Content

For detection of O_2_^•−^ fresh samples were thoroughly crushed in a 65 mM phosphate buffer pH 6.5. The homogenate was centrifuged at 12,000× *g* for 15 min at 4 °C and obtained supernatant was mixed with 65 mM phosphate buffer pH 7.8. The diluted supernatant was then mixed with 10 mM hydroxylamine hydrochloride and incubated 30 min at 25 °C. After that 7 mM alfa-napthylamine and 10 mM sulfanilamide were added and kept at 37 °C for 20 min. From mixture O_2_^•–^ was quantified according to Ghosh et al. [23].

### 2.8. Quantification of Total Capsaicin Content

In this case, 500 mg of dried fruits from each replicate crushed into fine powder and extracted with 10 mL of acetone for 5 h. Then all samples centrifuged at 10,000× *g* and supernatant collected. 4 mL of supernatant for each sample were taken into separate test tube and the capsaicin content was determined at 660 nm according to Sadasivam and Manickam [24]. Capsaicin content is expressed in µg capsaicin g^−1^ dry weight (µg g^−1^ DW).

### 2.9. Quantification of Polyamine and TLC Separation

500 mg of fruit tissue was crushed in 10% perchloric acid and homogenates were reacted with dansylchloride and run through silica TLC plate and quantified according to Wettlaufer and Weinstein [25].

### 2.10. Enzyme Activity Assay

#### 2.10.1. Esterase (EC 3.1.1.1) Activity

One g of fruit pericarp tissues was frozen in liquid N_2_ and ground into fine powder. From the powdered tissue, esterase activity was measured according to Kumar et al. [26].

#### 2.10.2. Polyamine Oxidase Activity and Diamine Oxidase Activity

Fruit samples were frozen with liquid N_2_ and crushed into powder for enzyme extraction. The enzyme extraction and activity were determined for polyamine oxidase activity (PAO; EC 1.5.3.11) and diamine oxidase activity (DAO; 1.4.3.22) according to Moschou (2018), respectively.

### 2.11. Extraction of Essential Oil

Fifty grams of fruit from each treatment including control were chopped into manageable pieces and gone through hydro distillation by clevenger type apparatus [27]. Then essential oil was extracted by dichloromethane (DCM) and condensed with a rotatory evaporator. Before GC MS analysis DCM fraction of essential oil was dehydrated over anhydrous sodium sulphate.

### 2.12. GC-MS Analysis of Essential Oil

GC-MS analysis was carried out using Agilent 7000 (Agilent Technologies Inc., Cheadle, UK) and compounds were separated using Agilent 122-5532 DB–5MS column. Helium was used as a carrier gas with a flow rate of 1 mL min^−1^ and injection volume was 2 µL and slit injection was used with ratio of 10:1. The inlet temperature was 250 °C and the ion source temperature 230 °C. Total run time was 60 min and the range of mass scanned was 50 to 550 amu GC-MS data were analyzed with Chem-Station (Agilent Technologies Inc., Cheadle, UK) following identification of compounds from retention index and custom mass spectra with NIST, 2014 library. Retention indices were calculated using n-alkane series C7 to C40 under sample operating parameter. Experiment was carried out with two replicates.

### 2.13. Characterization of TiO_2_-NP

The morphology, size and atomic composition of TiO_2_-NP was determined by SEM (GeminiSEM 450, Zeiss, Oberkochen, Germany) equipped with Energy Dispersive X-ray (EDX) module according to Femi-Adepoju et al. [28]. Determination of functional group was carried out Fourier transform infrared spectroscopy (FTIR, Agilent Cary 630, Agilent Technologies India Pvt Ltd, Delhi, India) analysis with spectral scan od 450–4000 cm^−1^ at a resolution of 4 cm^−1^ [29].

### 2.14. Statistical Analysis

All the acquired data were analyzed by Student’s *t*-test (SPSS, IBM, Armonk, New York, NY, USA) for analysis of the difference between treatments. The statistically significant differences were considered at *p* ≤ 0.05. The obtained data were presented at mean ± standard error (SE). The correlation figure was created through software.

## 3. Results

### 3.1. Firmness, Color, Turgidity and Total Soluble Solids of Fruit on TiO_2_-NC Treatment

**Figure 1:**
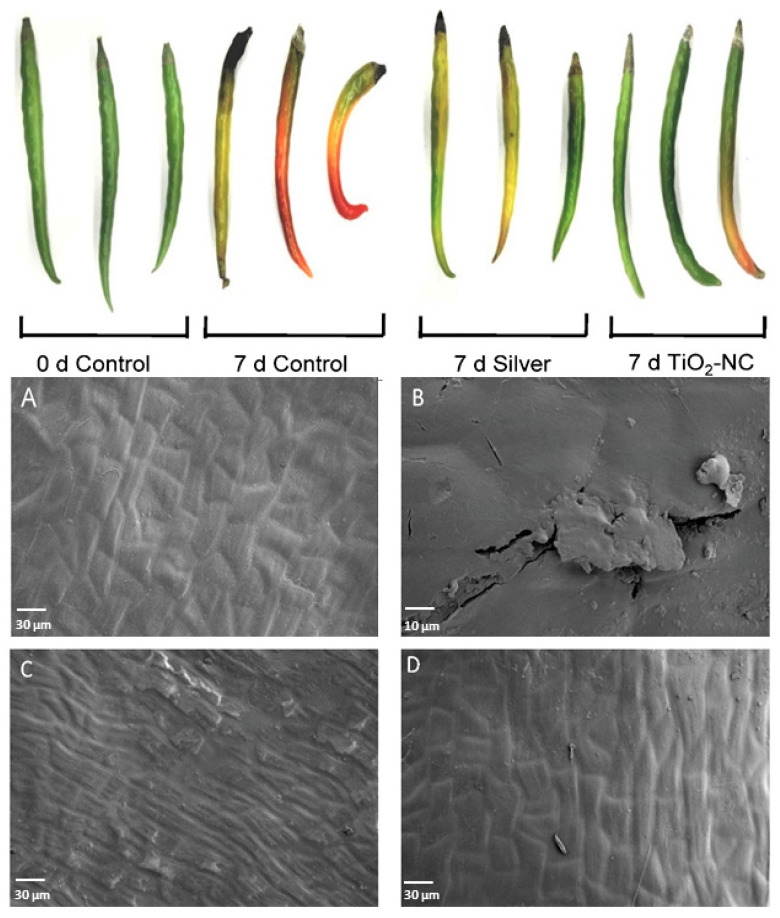
Changes in morphology and changes in fruit coat surface as revealed through scanning electron micrograph (SEM) in *Capsicum* fruit. The SEM images revealed the microscopic cracks and crevices on fruit coat surfaces and their variations on 7 d (**B**) postharvest storage over 0 d control (**A**). The 7d silver (**C**) and 7d TiO_2_-NC treatments (**D**) had their respective impacts on the possible repair of cell wall lysis.

**Figure 2:**
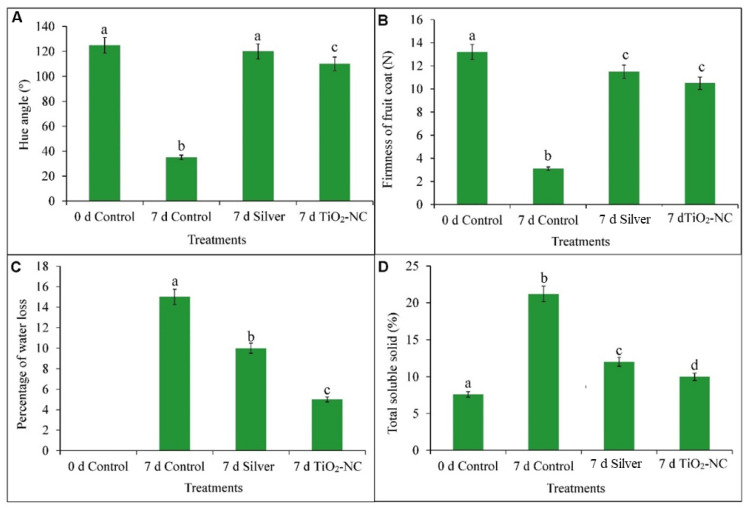
Hue angle (**A**), fruit firmness (**B**), percentage of water loss (**C**) and total soluble solid (**D**) of *Capsicum* fruit recorded under silver and TiO_2_-NC with respect to control during 0 d and 7 d of postharvest storage at 25 °C. Vertical bars showing SE± 1 (*n* = 3) on each column and letters above bars indicate statistical variations between treatments based on One Way ANOVA (*p* ≤ 0.05).

Developmental changes in fruit 7 d of ripening period under storage recorded significant morphological variations (Figure 1). The changes in color, texture, fruit coat firmness was notably affected with treatments such as silver or TiO_2_-NC (Figure 2). The surface morphology as critically analyzed by SEM studies for fruit coat which recorded a distinct variation through the treatments (Figure 1). Interestingly, the surface was significantly affected with 7 d on postharvest storage indicating the changes through ripening activities including loss of water. This pledges a possible relationship with other qualitative characters for ripening on fruit. This exclusively shows distinct crack and crevices on the cell wall that might ooze out cell sap and odor as a mark of ripening, however, in general. This feature was almost erased when fruit were observed from either silver or TiO_2_-NC, still maximum for the latter. Remarkably, the changes on cell surface nearly exist in parallel with variations in fruit color through the days of storage. Therefore, a possibility may arise on recovery of any cellular lysis of fruit coat being quite usual as usual for ripening under the aid of TiO_2_-NC.

The changes in fruit color as measured by hue angle had significant variation under both treatments (Silver and TiO_2_-NC) compared to control at 7 d of storage. Both silver and TiO_2_-NC maintained the color with only 4 and 12% decrease in hue angle compared to 72% in 7 d control over control at 0 d (Figure 2A). The firmness of fruit in the pressure unit (N) reveals the integrity of *Capsicum* fruit coat when observed under different treatments. A significant (*p* ≤ 0.05) reduction by 76% was recorded from fruit on 7th d of storage as compared to the initial day (Figure 2B). Interestingly, silver and TiO_2_-NC maintained firmness with only 13 and 20% reduction with respect to control at 0 d. The individual interaction of silver and TiO_2_-NC showed no significant (*p* ≤ 0.05) variations infirmness of fruit at 7 d.

With the duration of storage time fruit also observed gradual dehydration or loss of seeming freshness. This was documented with the percentage of water loss by gravimetric method and maximum loss of water was recorded in 7 d control by 15% compared to 0 d control (Figure 2C). Whereas, both silver and TiO_2_-NC had proportionately reduced water loss by 50 and 100%, respectively, over control at 7 d. On the other hand, fruit accumulated soluble solid that varied significantly through the treatments. (Figure 2D). The maximum amount of TSS was recorded in 7 d control which accounts 76% increase over 7 d silver. The treatment such as silver and TiO_2_-NC significantly (*p* ≤ 0.05) reduced TSS accumulation in respective to 7 d control. Still, TiO_2_-NC had reduced the content by 47% over control (7 d) and thus suggested more effectiveness in delayed ripening with a negative correlation value (r = −0.97) with the firmness of the fruit coat.

### 3.2. Absorption of Ti and Release of Ethylene from Fruit Tissues

**Figure 3:**
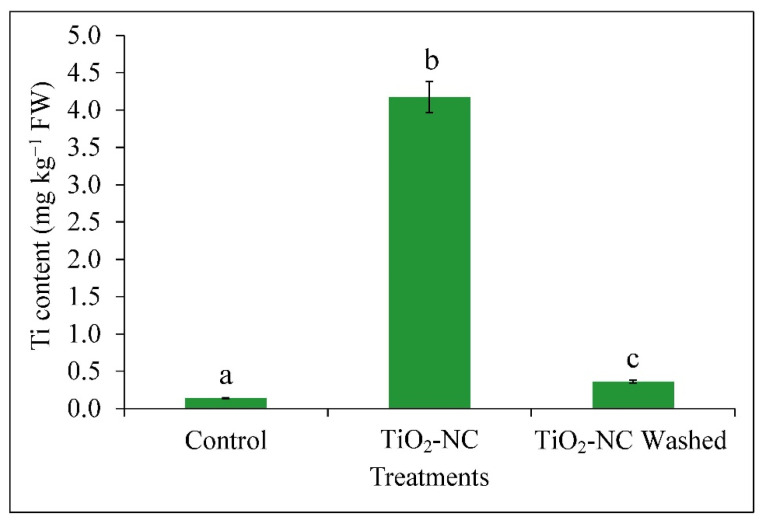
Absorption of Ti in *Capsicum* fruit recorded from 7 d TiO_2_-NC treatment and 7 d TiO_2_-NC washed with respect to control on 7 d storage under 25 °C. Vertical bars showing SE ± 1 (*n* = 3) on each column and letters above bars indicate statistical variations between treatments based on one way ANOVA (*p* ≤ 0.05).

The biosorption of the Ti within the fruit tissues was interesting when observed after 7 d of TiO_2_-NC treatment compared to control (untreated). In one of those groups treated (TiO_2_-NC) fruit, metal content was directly measured (Ti) without any washing and these samples recorded 4.17 mg kg^−1^ DW which was only 0.14 mg kg^−1^ DW in untreated control (Figure 3). In another group, samples were thoroughly washed, dried and measured the metal content show a significantly (*p* ≤ 0.05) lower values, an almost negligible increase of 0.22 mg kg^−1^ DW over control. Thus, as on a comparative basis, it accounted for 91% reduction of Ti over TiO_2_-NC before washing. This implies a significant low absorbing trend of Ti within the fruit tissues when applied with chitosan amalgamation as TiO_2_-NC. Therefore, the application of TiO_2_-NC may involve the phenomenon of biosorption of metal rather than bioaccumulation which may have entered into the fruit tissue through opening present is pedicel region of fruit. This may recommend TiO_2_-NC would be a safe residue to be applied as a postharvest preservative for its non-invasive nature.

**Figure 4:**
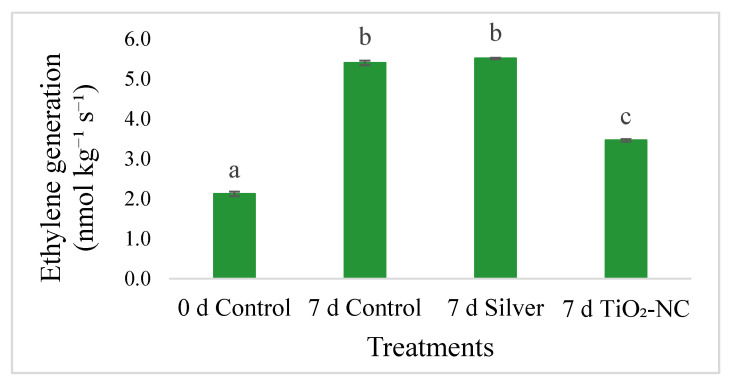
Changes in ET evolution of *Capsicum* fruit under silver and TiO_2_-NC with respect to control during 0 d and 7 d of postharvest storage at 25 °C. Vertical bars showing SE ± 1 (*n* = 3) on each column and letters above bars indicate statistical variations between treatments based on one way ANOVA (*p* ≤ 0.05).

Endogenous ET in the fruit tissues was recorded in varying concentrations from different treatments. Thus, control fruit at 7 d recorded significant upregulation of ET generation against 0 d control and measured 5.4 nmol kg^−1^ s^−1^ compared to 0 d control 2.1 nmol kg^−1^ s^−1^ (Figure 4). The treatments such as silver or TiO_2_-NC had significant variations in ET content as expected over the control. So, the release of endogenous ET was maximized under silver treatment with 83% increase from 7 d TiO_2_-NC but not with significant variation against 7 d control (Figure 4). On the contrary, the ET content was downregulated by 36% under the same condition when fruit were treated with TiO_2_-NC (Figure 4). The changes in ET concentration were significantly impacted on quality characters TSS (r = 0.75), firmness (r = −0.62), hue angle (r = −0.54) with as evident from the correlation matrix (Supplementary Materials Table S1).

### 3.3. Expression of Genes for Ethylene Biosynthesis and Signaling

**Figure 5:**
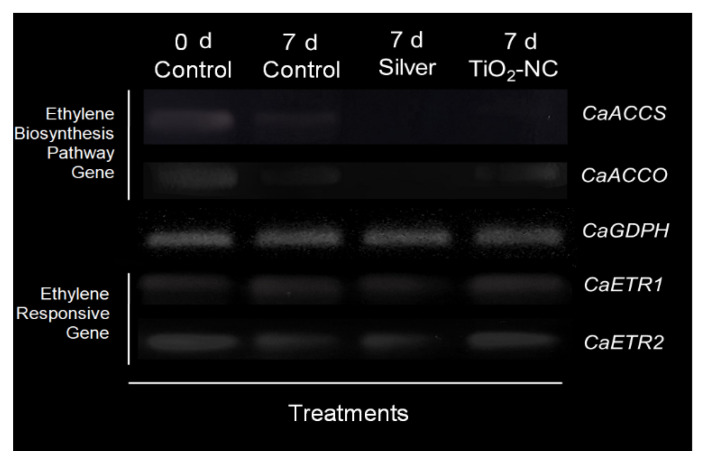
Expression of *CaACCS*, *CaACCO*, *CaETR1*, *CaETR2* genes as compared to *CaGAPDH* as control gene in *Capsicum* fruit under silver and TiO_2_-NC with respect to control during 0 d and 7 d of postharvest storage at 25 °C.

Expression levels of the targeted homologs for transcripts for ET biosynthesis and signaling genes, viz., Ca*ACC synthase*, Ca*ACC oxidase* and Ca*ETR1* and Ca*ETR2* were checked. Through the semi-quantitative RT-PCR technique, the transcripts were evaluated against Ca*GAPDH* (as internal control) and those revealed about downregulation for *ACC synthase* under TiO_2_-NC against 0 d control. During storage of 7 d, the expression changed under the same treatment which was significantly (*p* ≤ 0.05) higher than silver (Figure 5). Meanwhile, silver had changed the expression of a gene compared to respective 0 and 7 d control. For conversion of ACC to ET the gene involved as Ca*ACC oxidase* had inclined a similar pattern of expression. However, for both the treatments such as silver and TiO_2_-NC expression was more pronounced by distinct suppression respective genes (Figure 5). Contrarily, signaling pathways of ET governed by different genes: Ca*ETR1* and Ca*ETR2* had distinct variations in expression under treatment concerned. Interestingly, for *ETR1* expression was quite abundant by transcript densities of 7 d control less than TiO_2_-NC under same duration (Figure 5). Thereafter, the variations were more pronounced by 25% downregulation under silver and upregulation under TiO_2_-NC over the 0 d control. For other genes such as Ca*ETR2*, the relative expression recorded a steady fall from ongoing days of storage under 7 d control (Figure 5). Interestingly, TiO_2_-NC had retrieved the expression of *CaETR2* as compared to silver. Still, silver was not able to upregulate *ETR2* as TiO_2_-NC rather subdued the expression on completion of storage i.e., after 7 d.

### 3.4. Accumulation of ROS (H_2_O_2_ and O_2_^•−^) in Fruit Tissues under Storage

**Figure 6:**
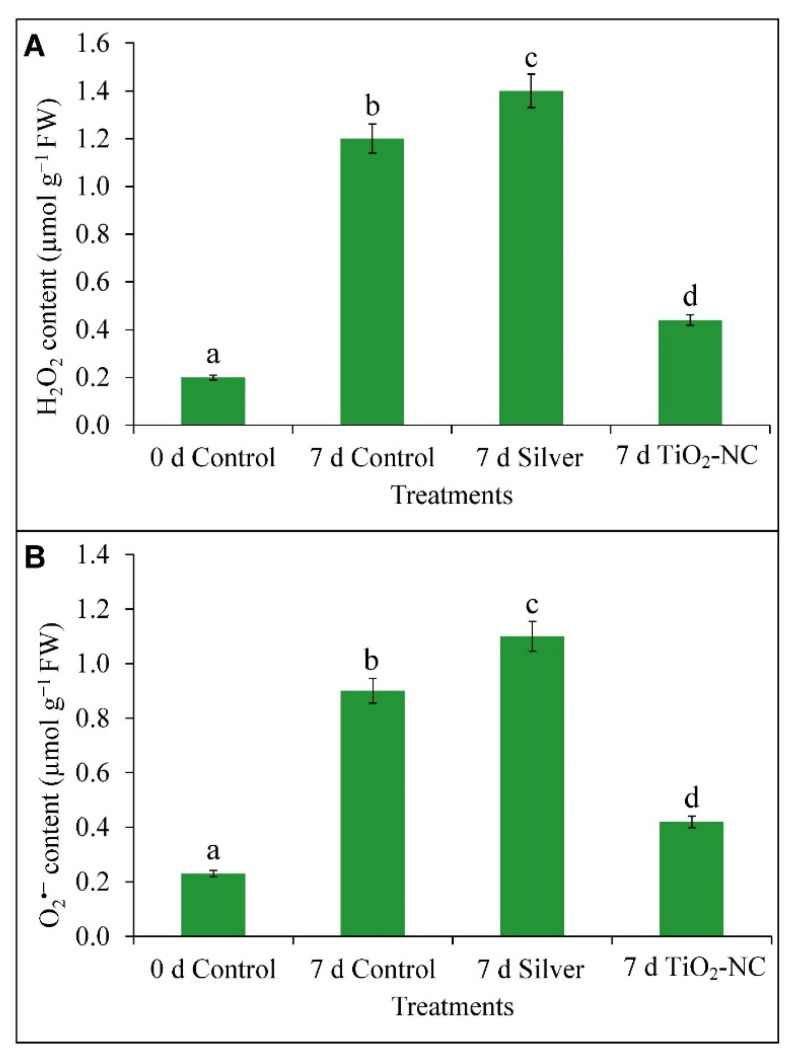
H_2_O_2_ content (**A**) and O_2_^•−^ content (**B**) of *Capsicum* fruit recorded under silver and TiO_2_-NC with respect to control during 0 d and 7 d of postharvest storage at 25 °C. Vertical bars showing SE ± 1 (*n* = 3) on each column and letters (a, b, c and d) above bars indicate statistical variations between treatments based on one way ANOVA (*p* ≤ 0.05).

H_2_O_2_ and O_2_^•−^ followed almost a similar pattern in control and treatments up to the completion of the storage period. In the case of H_2_O_2_, the maximum accumulation occurred (17% over 7 d control) in fruit tissue when observed from silver treatment. Conversely, TiO_2_-NC reduced the content on 7 d storage which recorded 63% less than 7 d control (Figure 6A). TiO_2_-NC had suppressed O_2_^•−^ accumulation by 69% over the silver under the same condition (Figure 6B). Fruit proceeding through storage period recorded 0.67 µmol g^−1^ increase in O_2_^•−^ content, however, under control condition. Furthermore, treatments such as silver had the impact on ripening to accumulate O_2_^•−^ by 0.87 µmol g^−1^ over 0 d control. On the contrary, TiO_2_-NC rather reduced the ROS (O_2_^•−^) significantly (*p* ≤ 0.05) by 53% in contrast with silver as compared to 7 d control.

### 3.5. Catabolism of Polyamine during Ripening on Storage

**Figure 7:**
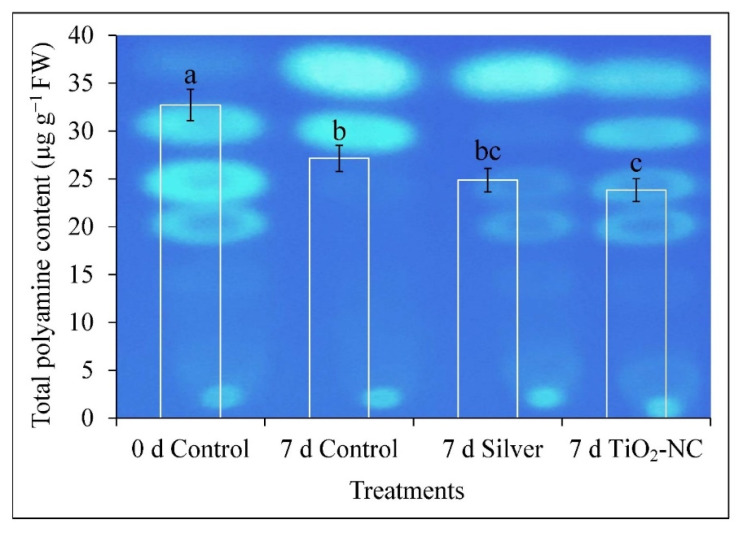
Separation and identification of total polyamine from *Capsicum* fruit on TLC plate and resolved under UV illumination under silver and TiO_2_-NC with respect to control during 0 d and 7 d of postharvest storage at 25 °C. Vertical bars showing SE ± 1 (*n* = 3) on each column and letters above bars indicate statistical variations between treatments based on one way ANOVA (*p* ≤ 0.05).

Ripening of fruit with many physiological effects by ET intervention are antagonized by polyamines. As Figure 7 shows the total polyamine content gradually decreased during the ongoing storage period. This decrease was maximum at TiO_2_-NC which shows 27% decrease with respect to 0 d control at 7 d. Increased polyamine often supports fruit firmness, redox maintenance, sugar metabolism those all for the prevention of ripening. Therefore, turnover of polyamines is likely to be hastening the fruit senescence, specifically through ROS generation under storage conditions.

**Figure 8:**
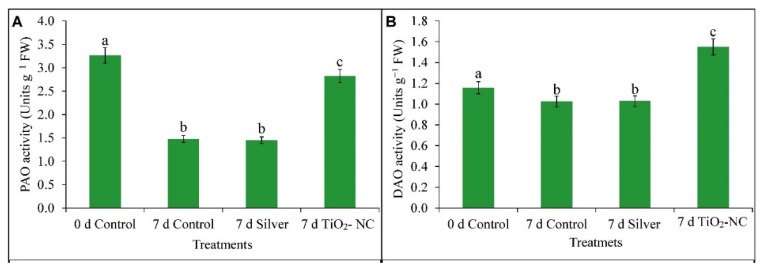
Activity of PAO (**A**), DAO (**B**) from pulp tissues of *Capsicum* on tissues under silver and TiO_2_-NC treatments with respect to control (0 d) and 7 d storage of postharvest at 25 °C. Vertical bars showing SE ± 1 (*n* = 3) on each column and letters (a, b, c and d) above bars indicate statistical variations between treatments based on one way ANOVA (*p* ≤ 0.05).

On this notion, we checked the catabolism of polyamines by enzymatic lysis with polyamine oxidase activities. This is more to predict the correlation (*p* ≤ 0.05, *r* = −0.99) of ROS (H_2_O_2_) generated by polyamine oxidation and its contribution to the peroxidation of fruit. Thus, the activity of DAO enzyme had the downregulation through 7 d storage, however, under control by 11% over the initial (Figure 8B). Treatment such as silver or TiO_2_-NC had their respective effects on DAO activity. Thus, under silver treatment, the subdued activity recorded 11% less over control whereas, it was upregulated by 33% for TiO_2_-NC (Figure 8B). An almost similar or compatible trend was noticed for PAO also where fruit attained a peak activity at 0 d control. Two treatments such as silver and TiO_2_-NC were more capable to minimize the activities of the enzymes where 64 and 14% fall in values over the 0 d control were recorded during storage (Figure 8A). Although PAO activity is higher in TiO_2_-NC with respect to other treatments at 7 d, lower polyamine content in fruit tissue may have minimized its impact on fruit ripening.

### 3.6. Esterase Activity and Its Polymorphic Expression on Fruit Softness

**Figure 9:**
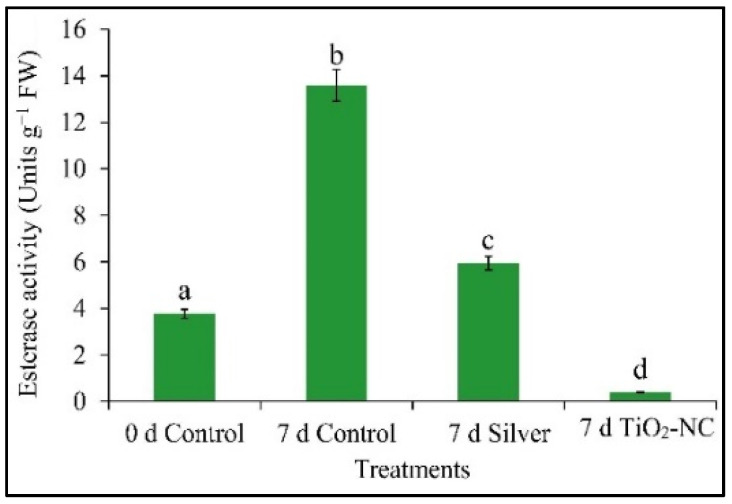
Activity of esterase from pulp tissues of *Capsicum* on tissues under silver and TiO_2_-NC treatments with respect to control (0 d) and 7 d storage of postharvest at 25 °C. Vertical bars showing SE ± 1 (*n* = 3) on each column and letters above bars indicate statistical variations between treatments based on one way ANOVA (*p* ≤ 0.05).

Changes in the functioning of various cell-wall bound or cytosolic hydrolases including esterases are important in fruit softening. Ongoing days of postharvest storage *Capsicum* fruit recorded a distinct variation of esterase activity in 7 d silver and 7 d TiO_2_-NC treated fruits with respect to 7 d control. From the initial (0 d) period of ripening fruit showed a sharp rise of esterase activity by 10 units reaching at 7 d, however, at control. Still, on the activity of silver and TiO_2_-NC the activity of esterase reduced by 56 and 96% over control (Figure 9). Interestingly, the activity of esterase under those treatments had an impact on fruit firmness with a significant (*p* ≤ 0.05) effect as reading correlation matrix. The influence of TiO_2_-NC for its altered activity had also been accompanied by polymorphic expression of esterase at the gene level. Thus, esterase profile may set as a possible biomarker as *Capsicum* fruit attained the delayed ripening under silver or TiO_2_-NC.

### 3.7. Changes in Essential Oil Contributing Major Metabolites in Ripening

**Figure 10:**
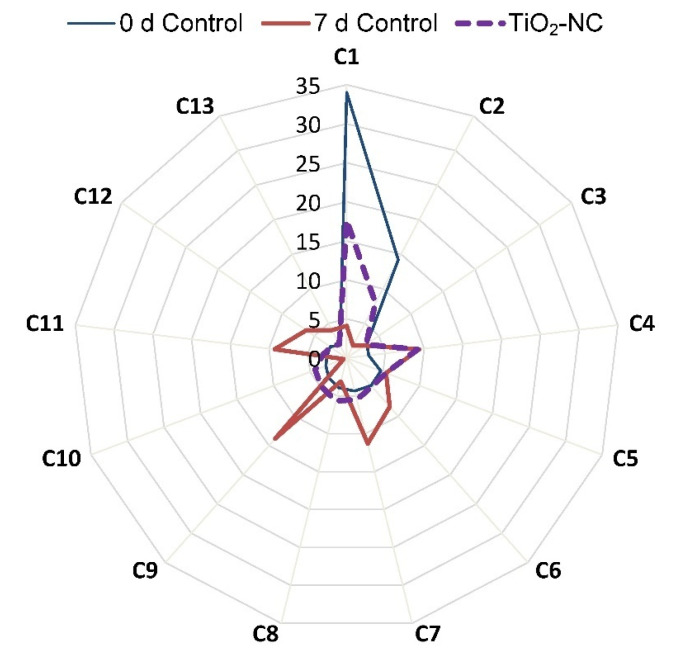
Radar plot showing the pattern of changes in concentration of major constituents of essential oil under TiO_2_-NC treatments with respect to control at 0 d and 7 d storage of postharvest at 25 °C.

The major metabolites were separated, resolved their identification and relative concentration were determined with GC-MS studies. From overall 52 identified metabolites, it was only 13 had significant up/downregulation in the content through the storage period which includes alkane, ester, fatty acid and alcohol etc. (Table 1). It is quite clear from the Figure 10 constituents of essential oil follow a pattern which indicated ongoing ripening process. On specific constituent of essential oil such as C1 (Cuben-11-ol), C2 (Spiroether) and C6 (1,3-dihydroxypropane-2-ylhexadecanoate) it exhibited sharp decrease concentration by 30, 12 and 0.4% at 7 d storage, whereas, it is only 16, 6 and 0.13%, respectively, under influence of TiO_2_-NC over the 0 d control (Figure 10). Contrarily, alkanes such as C9 (Heptacosane), C11(Triacontane), C12 (Hentriacontane) recorded significant increase at 7 d control by 10, 7 and 3.21%, respectively over 0 d control. On the other hand, TiO_2_-NC treatment recoded just 44 and 21% increase C9, C11, respectively, and C12 decrease by 0.09% compared to control (Table 1).

### 3.8. Capsaicin Content

**Figure 11:**
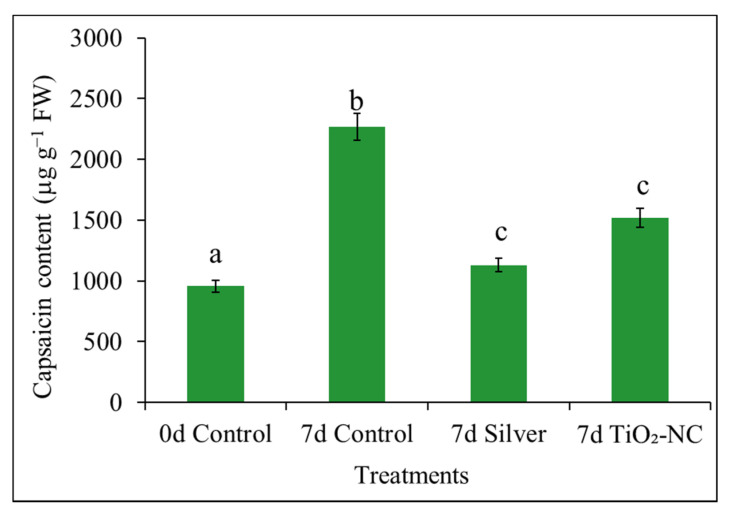
Capsaicin content of *Capsicum* fruit recorded under silver and TiO_2_-NC with respect to control during 0 d and 7 d of postharvest storage at 25 °C. Vertical bars showing SE ± 1 (*n* = 3) on each column and letters above bars indicate statistical variations between treatments based on one way ANOVA (*p* ≤ 0.05).

Capsaicin content was quantified spectrophotometrically which is main contributing factor for pungency. It has been observed that capsaicin content increase as the fruit ripening during storage (Figure 11). Likewise, capsaicin content increased by 1312 µg kg^−1^ at 7 d control with over 0 d control. On the other hand, capsaicin content of 7 d silver and 7 d TiO_2_-NC increase by 15 and 59% over control at initial day. Changes in the capsaicin content showed positive correlation with ET content and negative strong correlation with hue angle and firmness of fruit.

### 3.9. TiO_2_-NP and Its Opto-Physico-Chemical Characterizations

**Figure 12:**
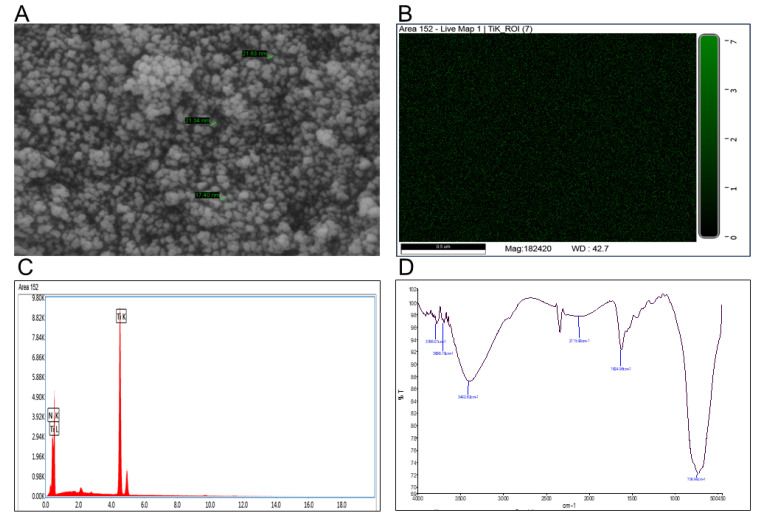
SEM image showing size dstribution of TiO_2_-NPs (**A**), EDAX map TiO_2_-NPs to confirmtion the presence of Ti (**B**), quantification of Ti by EDAX in the TiO_2_-NP (**C**) and FTIR showing distribution of different bonds present in TiO_2_-NPs (**D**).

As described earlier molecular nature of TiO_2_-NP was confirmed by different physico-optical properties. This assumed the uniform sizes of particles as bearing conformity with earlier description [29]. Preliminarily, FE-SEM study was carried out for the shape and size of nanomaterial. This revealed diameter of NP ranges between 15–30 nm which was within the limit of ≤100 nm size as resolved in SEM image and appeared spherical to the globular unit (Figure 12A). From the SEM EDAX the presence of Ti in the NP has been verified which is represented as green dots in Figure 12B and it also shows a sharp pick of Ti in Figure 12C which confirms the presence of Ti in NP. The FTIR spectroscopy finally revealed the associated bond energy changes (cm^−1^) from projected varying functional groups of TiO_2_-NP (Figure 12D) [30]. Largest pick was observed at 726.96 cm^−1^ along with another small pick at 482 cm^−1^ which confirms presence of characteristic O-Ti-O bond in TiO_2_-NP. Other picks at 3402.82 cm^−1^ and 1624.96 cm^−1^ indicated surface absorbed water and hydroxyl bonds, respectively (Figure 12D).

### 3.10. Correlation among the Parameters

**Figure 13:**
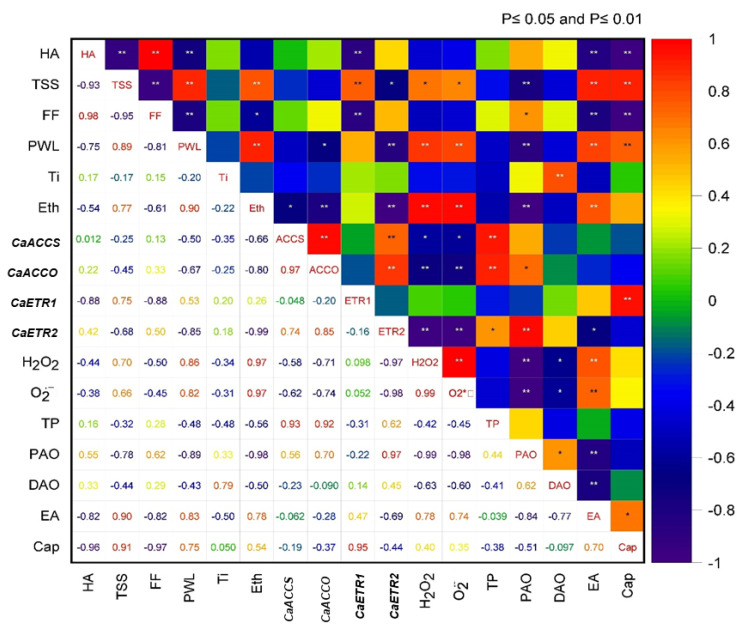
Correlation matrix heatmap of significant attributes in modulation ripening process under treatments. Here, lower triangle represents correlation values and upper triangle represents the significance levels (* and ** represent significance level at *p* ≤ 0.05 and *p* ≤ 0.01, respectively) between different parameters. HA-Color in hue angle, TSS-Total Soluble Solid Content, FF-Firmness of fruit coat, PWL-Percentage of water loss, Ti-Titanium accumulation, Eth-Ethelene content, ACCS-expression of *ACC Synthase*, ACCO-Expression of *ACC oxidase*, ETR1-Expression of *ETR1*, ETR2-expression of *ETR2*, H_2_O_2_-Hydrogen peroxide content, O_2_^•−^-superoxide content, TP-Total Polyamine Content, PAO-Activity of PAO, DAO-Activity of DAO, EA-Activity of Esterase, Cap-Capsaicin content.

From the Pearson’s correlation matrix, it comes in a note that physiological and cellular attributes in ripening are quite variable in contribution and more in a complex manner. Ripening of *Capsicum* fruit involves oxidative reactions, so ROS metabolism influencing ripening specific traits found significant, however, variably regulated under different treatment. Thus, initially changes in ROS (H_2_O_2_ and O_2_^•−^) related positively with ripening parameters such as TSS but negatively with firmness. Similarly, total polyamine content had a significant (*p* ≤ 0.05) negative correlation with ROS (Figure 13). The ROS-producing activity polyamine oxidation had a variable influence on ripening. Thus, DAO and PAO had an indirect contribution with ROS by the generation of H_2_O_2_ but not with others. On the other hand, ET accumulation with *ACC synthase* and oxidase had their respective values but not always directly contributed to ripening characters such as TSS, color, firmness etc. Contrarily, ET receptors, specifically *ETR2* had regulated negatively the ET accumulation thereby showing an indirect feedback on ripening. Moreover, signaling through *ETR2* had impacted in suppression of ROS such as both O_2_^•−^ and H_2_O_2_ content as observed through negative correlation values (Figure 13). So, ROS intervention in ripening phenomena is not simple but complex and discriminatorily in collusion with related physiological characters.

## 4. Discussion

### 4.1. Modulation of Quality Characters for Ripening under TiO_2_ Nanocomposite

Nanoparticles of titanium (TiO_2_-NPs) are less frequent in use but focus exclusively on their photocatalytic properties. Thus, TiO_2_-NPs in plants are exercised as a photocatalytic agent for ET with strong reducing activity under UV irradiation [31,32]. In postharvest storage of fruit and vegetables, the application of such an ET scavenger is thus entrusted more with its non-toxicity as well as less invasiveness within the fruit coats [33]. This have got evident in our experiment when *Capsicum* recorded a less bioaccumulation of Ti in the fruit pulp over the control. This is more realistic when we used the chitosan coating in the retention of hydration to support freshness during the storage of *Capsicum* fruit. Chitosan being semi-permeable in nature could modify the fruit tissue atmosphere, more specifically increasing hydration vis-a-vis regulating transpiration loss [23]. This makes it prudent for ET and its role in initiation, modulation and coordination of gene expression related to ripening. Therefore, initially for sensory characters such as color, firmness, relative water content etc. were more significantly bothered under treatment of TiO_2_-NP with chitosan (TiO_2_-NC) over silver treated samples. Stimulatingly, the experiment became optimistic in the delay of the ripening process when the TiO_2_-NC treatment sustained more firmness and color of the fruit through ongoing days of storage. This may be caused by combinational effect of chitosan (caused by inhibition of transpersonal loss of water and maintenance of fruit coat integrity) and TiO_2_-NP which reduce the ET accumulation by photocatalytic reductor process which cause delay in ET induced ripening responses such as turnover of pigments [34]. This assumes the possible suppression of ET in delaying of ripening process through other changes in the structural and functional integrity of fruit undergoing maturation. One of the most import characters of *Capsicum* is pungency which is mainly due to capsaicin, one of main metabolite in capsinoids family [35]. During postharvest storage capsaicin content becomes increased, which is an indication of progress of fruit ripening [36]. In this experiment TiO_2_-NC effectively decreased accumulation of capsaicin with respect to untreated control which is an indication of delayed ripening during postharvest storage. Interestingly, the TiO_2_-NC treatment was more lenient to maintain the fruit coat intactness as no significant rupture/scars were observed by SEM images as compared to silver-treated fruit. This ensures the acceptability of NC as ecofriendly chemical residues owing to non-invasive features. This is quite in agreement with previous cases where suppression of ET by 1-MCP had secured the firmness of fruit coat with a decrease in soluble solid content during storage [37]. The changes in soluble solid content are important for characterizing the ripening metabolism. Soluble solids cover heterogenous complex organic residues which in combination with titrable acidity make a reliable ripening index [38]. So, a decline or subdued of soluble solid concentration may serve the characteristics of delayed ripening. Thus, TiO_2_-NC would be reliable for maintaining the soluble solid throughout the postharvest circumvents the paths of retarded senescence. So, from the biosafety purpose *Capsicum* fruit in our experiment might acknowledge the efficacy of TiO_2_-NC in retention of firmness, color over refereed toxic of chemicals such as silver. Loss of water otherwise deducing the reduction of hydration or freshness of the fruit during postharvest storage had more success with TiO_2_-NC than silver. In general, chitosan, basically a cationic polymer of *N*-acetyl-d-glucosamine is served with an admirable film coating surface with hydrophilic activity. In addition to the hydrophilic activity of chitosan through semi-permeable film, it can control the tissue atmosphere decreasing the transpiration or vapor loss [19]. So, from fruit quality aspect application of chitosan with the combination of nanomaterial would be successful. Chitosan steeping with additional residues such as Ca^2+^ on fruit coat had impacted on key metabolites pool such as organic acid and its turn over governing moderation of shelf life in other fruit [39].

### 4.2. Transcript Dynamics of Ethylene Biosynthesis and Signaling Paths

Available information has emphasized the bioaccumulation of ET and its regulation is a key element for the moderation of postharvest ripening. Inhibitors such as 1-MCP not only played in the suppression of ET biosynthesis but also delay the fruit senescence through the accumulation of different antioxidants [40]. The latter in turn suppresses the membrane permeability as well as lipid peroxidation of the fruit coat. Herein also *Capsicum* fruit had significantly moderated under treatment of TiO_2_-NC as compared to standard ET action inhibitor such as metallic silver. The differential ET accumulation on storage is quite consistent for the expression of ripening specific genes such as Ca*ACC synthase* and Ca*ACC oxidase* [41]. This is quite satisfactory to receive a good regulation for both of those transcripts and their relative abundance under TiO_2_-NC. The transcriptional upregulation for ACC conversion would sensitize not only the ET accumulation but also induces gene cascade linked to other metabolites web. Therefore, ACC metabolism contributing ET moderation under any treatments would suffice the sustenance of greenness on storage where TiO_2_-NC might efficaciously qualify. ACC metabolic genes in nexus with anti-oxidation paths and their upregulation have maximum affinity when treated with blue LED irradiation as recoded in citrus [42]. This could be accounted in the present case when TiO_2_-NC with its s intrinsic property to be energized with UV irradiation which photocatalytic degrade ET. Inhibition of ET on the biosynthetic pool for delayed senescence a level of regulation exists with its downstream signaling for ripening genes. It is well agreed that ET signaling cascade is active in normal condition and binding of ET to its receptors facilitated autocatalytic degradation of certain regulatory elements of ET by feedback regulation. This contemplates for silver treatment is no longer with any inhibition for ET synthesis but may act as an inhibitor of the signaling pathway of ET sensitivity is based on activation of receptors such as *ETR2* and *EIN2* which at specific domain is occupied by silver in a competitive manner such as 1-MCP [41]. Ongoing days of ripening for *Capsicum* where TiO_2_-NC upregulated the transcriptional dynamics of *ETR1* and *ETR2* significantly over silver when compared to 0 d control. This establishes no regulation of TiO_2_-NC on action or functioning level besides exclusively for the subdued synthesis of ET by the compatible trend of *ACC synthase* and *ACC oxidase* expression. This is well consistent with the revealed fact from others’ studies in tomato, apple where treated fruit with 1-MCP had delayed the ripening both in terms of reduced ET biogenesis corresponding to the decrease in gene expression. However, non-inhibitory nature of TiO_2_-NC in ET signaling is otherwise advantageous over silver, however, in a different way. In fact, induction of Ca*ETR1* and Ca*ETR2* is otherwise involved for significant expression of related gene cascade of ET signaling. The latter includes the antioxidation-related gene in lysis of ROS; reduces peroxidation, carbonylation reactions and accelerates senescence, otherwise hastens ripening process. So, TiO_2_-NC may be more privileged over silver, 1-MCP such as conventional agents in the application for dual roles: to reduce the endogenous ET concentration and induce antioxidation activities to retards ripening in postharvest storage.

### 4.3. ROS Metabolism Is Related to Polyamine Oxidation in Progress of Fruit Ripening at Storage

Ripening being on penultimate stages of senescence characterized by hydrolysis and diverse peroxidative reactions on macromolecules with the generation of ROS [42]. It is with well evidential support that an active metabolism of ROS is involved in various species such as sweet pepper, tomato, guava, mango, grape etc. [43]. Consistently, the ROS metabolism and its antioxidation by nonenzymatic as well enzymatic paths characterize the changes in quality characters of fruit such as softening, color, odor. However, in-built antioxidation system or its improvisation by exogenous elicitors accommodated to regulate the ripening behavior within postharvest storage. Understanding the hastening of senescence by the advent of ROS fruit tissues in our experiment realized a significant moderation superoxide (O_2_^•−^), peroxide (H_2_O_2_) under TiO_2_-NC. ROS has its dual functionalities in ripening incident of fruit mostly with peroxidative lysis and activation of conserved ripening-related genes regardless of non-climacteric and climacteric types [44]. It is mostly the metabolism within mitochondrial energy-dependent paths and oxidation of few biomolecules connecting between ET production and respiratory during the ripening. The availability of ROS may also be circumvented by oxidation of residues polyamine being predominant. Polyamine, a ubiquitous polycationic straight-chain hydrocarbon residue with characteristics of few −NH_2_ functional groups with varied unsaturation exists in various cell fractions. This molecule undergoes protonated at biological pH and being nucleophilic in nature can bind most of the positively charged bioresidues in cells. This facilitates rendering a shielding effect from oxidation by ROS or free radicals under abiotic stress in plants [5]. In ripening fruit roles of polyamines are based on two facts: one, antagonizing the ET biosynthetic paths and shielding the biomolecules from peroxidative degradation by ROS developed from various catabolic reactions [45]. Polyamines use a common precursor (such as arginine) and biosynthetic paths with ET. This may be diverged depending on plants’ cellular demand from an intermediate such as ACC and directed either into polyamine or ET. Postharvest period triggering more ET biosynthesis than polyamine is obvious to expedite the fruit senescence. A consistent inverse interrelationship has been common in many cases between ET and polyamine metabolism in ripening fruit. Thereby, a depletion of polyamine on 7 d of storage in *Capsicum* fruit might link the increased activities of DAO and PAO under treatment of silver and TiO_2_-NC in favor of more ROS such as H_2_O_2_ [46]. H_2_O_2_, however, within a threshold concentration, in turn, be inductive of several antioxidation cascade-like activities of class III peroxidase to reduce the oxidative damages of fruit tissues and thereby impeding the ripening process.

### 4.4. Metabolites Profiling: The Abundance of Essential Oils Induced by Nanocomposite

The ripening is highly regulated by abundance of specific metabolites influencing quality characters. Instead of a single one metabolite collection of different metabolites have wide coverage through ripening period as well as proportionate contribution to agronomic values [47]. In *Capsicum* the development of essential oils with ripening progress happens to be a good correlative index with few characters (Supplementary Materials Table S1). This finding would be important and contested for ripening specificity of fruit where mostly alkane group of residues were synchronously increased [48]. On contrary the alcohols and esters were also documented in a negative manner for contribution on ripening specific changes. Therefore, conclusively essential oil constituents with special reference to alkane residues can be considered as screening indices for delayed ripening *vis-à-vis* extended shelf life of fruit on postharvest storage. Those such as color, texture, softness etc. necessarily indicative of ripening stages by significant changes with up/downregulation. Essential oils its constituent residues are associated with capsinoids in support of hot favor and intense burning sensation for pepper consumption. Factually, *Capsicum* fruit from many cultivars have been frequently in more exercise for its essential oils and adjoining compounds (phenolics, terpenoids, steroids) than that of capsinoids also. In the contribution of specific aroma pepper fruit with their volatile oils, however, relatively low in concentration may vary depending on harvest stages and supplementation of agronomic inputs [49]. The chief constituents of pepper oil and related residues cover esters, small chain fatty acid, alcohol, ketones, unsaturated hydrocarbons, benzene and naphthalene derivatives, sulfur residues etc. This is interesting to note that *Capsicum* fruit are well responsive to TiO_2_-NC in upregulation of essential oils, however, few of those. This would be more imperative having a significant correlation with any ripening-related phenomena, particularly, in the contribution of quality characteristics. Identification of several hundred constituents in essential oils as percentage varied from dry and fresh fruit categorically grouped into aldehyde, terpenoid, hydrocarbon. Additionally, ketone, alcohol, unsaturated/saturated fatty acids, esters are adjoining to complement the physicochemical properties of essential oil [50].

Contextually, metabolites flux as studied in GC-MS analysis in the *Capsicum* fruit and its comparative allocation through ongoing days of ripening in the present experiment is interesting. As compared to initial days of ripening fruit recorded a significant metabolites allocation variably with silver and TiO_2_-NC. In general fruit ripening being a penultimate stage of senescence is characteristically experienced with both re-synthesis and turn of metabolites. Maximum accessible research has been attentive on analyzing the organic acids, secondary metabolites, sugar residues, volatile compounds and changes of their anabolic and catabolic paths. However, in pepper fruit residues are in different proportions as principle depending on stages of maturity [51]. It also identifies terpene(s), esters, ketones, naphthalene, phenolics, hydrocarbon, aromatic volatiles etc. This may establish the metabolic imprint that necessarily validates the role(s) of specific compounds satisfying paths of ripening under TiO_2_-NC. Expectedly, this makes it prudent to suffice the biomarker concept through the exhibition of ripening specific residues matching with existing GC-MS library attributing regulation of postharvest shelf life of *Capsicum*. Identified residues in essential oils varied under TiO_2_-NC may not be any exceptions in quality but in quantity as compared to control. The changes in content according to storage would be rather significant otherwise. It would be overbearing to focus any specific residues if expressed in parallel to definite any quality characters for in moderation of ripening. It offers a selective pressure for *Capsicum* fruit for delayed senescence based on such a metabolomic profile of essential oil. In pursuit of overexpression of such a metabolite(s), identification of key/regulatory gene(s) for its biosynthetic paths, would be the target in better postharvest management.

## 5. Conclusions

This work is first time report on effect of TiO_2_-NC for its biocompatibility and moderation of ripening phenomena on postharvest storage at 25 °C. The exercise of a bio safe and non-toxic TiO_2_-NC (since its concentration is insignificant in fruit tissues) in our experiment with *Capsicum* fruit had evident it to be potential. This is proved exclusively in ripening stipulated cellular responses attributing ET metabolism as studied herein. The spotlight of the experiment on photocatalytic break down of endogenous ET and changes signaling by TiO_2_-NC had verified the potentials of ripening postpone by ROS metabolism. It moderated the ET biosynthetic (*CaACCS* and *CaACCO*) as well as signaling regulatory (*CaETR1* and *CaETR2*) genes in parallel to changes the sensory characters in fruit. The moderation of ET signaling had carry forwarded in the upregulation of polyamine-mediated ROS generation during storage. The enzymatic oxidation of polyamines by DAO and PAO and changes had fairly characterized ripening during storage. TiO_2_-NP had received attention in the identification of metabolite flux contributing to the moderation of any ripening volatile residues. A distinct variation of those constituents under influence of TiO_2_-NC is a pertinent feature for any change in ripening under treatments. This generation of metabolites could serve as a biomarker in support of delayed senescence, however, directly or indirectly. As a whole, this study verified the nature of biological interaction for TiO_2_-NC on *Capsicum* fruit for alteration of ripening during postharvest storage. Still, it awaits deep insights for underlying mechanism or reactivity in favor of TiO_2_-NC perception to peruse other specific paths of ripening of fruit.

## Figures and Tables

**Table 1:** Major composition of essential oil in *C. annuum* fruit under various treatments.

No.	Name of Compound	Calculated KI	Reported KI	Concentration (%)		
				0 d Control	7 d Control	TiO_2_-NC
C1	Cuben-11-ol	1595	1595	34.02	4.12	17.66
C2	Spiroether	1880	1879	14.22	1.78	7.94
C3	1,3-dihydroxypropan-2-ylhexadecanoate	2289	2289	3.15	2.79	3.02
C4	Methyl labdanolate	2382	2381	2.83	9.36	9.11
C5	Tetracosane	2388	2400	4.67	5.39	5.40
C6	Pentacosane	2488	2500	4.74	8.32	4.88
C7	Hexacosane	2588	2600	4.36	11.30	5.50
C8	Nonacosanoicacid	2673	2674	3.95	3.08	5.65
C9	Heptacosane	2745	2700	3.41	13.79	4.90
C10	Pentatriacontane-2-one	2873	2873	2.85	0.40	4.31
C11	Triacontane	2985	3000	2.50	9.31	3.10
C12	Hentriacontane	3084	3100	2.55	6.26	2.46
C13	Dotriacontane	3183	3200	1.84	4.02	2.02
Total percentage				85.10	79.93	75.94

## Data Availability

All data are available in this manuscript.

## Supplementary Materials

The following supporting information can be downloaded at: https://www.mdpi.com/article/10.3390/plants11040513/s1, Figure S1: *CaERT1* and *CaETR2* gene expression bands under different treatments with 100bp DNA marker; Table S1: Correlation matrix of significant constituents of essential oil and quality indices in modulation ripening process under treatments; Table S2: Primer used in the PCR reaction.
