# Supplementary material for: Photoactivated TiO2 Nanocomposite Delays the Postharvest Ripening Phenomenon through Ethylene Metabolism and Related Physiological Changes in Capsicum Fruit
Source: Plants (Basel). 2022 Feb 14;11(4):513. doi: 10.3390/plants11040513 (PMC8876699; doi:10.3390/plants11040513)
Supplement: Supplementary file 1 [file plants-11-00513-s001.zip › plants-1543831-supplementary.pdf]

Table S1: Correlation matrix of significant constituents of essential oil and quality indices in modulation ripening process under treatments

	HA	FF	PWL	TSS	C1	C2	C3	C4	C5	C6	C7	C8	C9	C10	C11	C12	C13
**HA**	1	***	***	***	***	***	***	*	*	***	***	*	***	***	***	***	***
**FF**	0.994	1	***	***	***	***	***	**	**	***	***		***	***	***	***	***
**PWL**	-0.984	-0.997	1	***	***	***	***	**	**	***	***		***	**	***	***	***
**TSS**	-1.000	-0.995	0.986	1	***	***	***	*	*	***	***	*	***	***	***	***	***
**C1**	0.912	0.950	-0.970	-0.916	1	***	***	***	***	***	***		***		***	***	***
**C2**	0.931	0.964	-0.981	-0.935	0.999	1	***	***	***	***	***		***	*	***	***	***
**C3**	0.977	0.994	-0.999	-0.979	0.979	0.988	1	***	**	***	***		***	**	***	***	***
**C4**	-0.655	-0.730	0.778	0.662	-0.907	-0.885	-0.802	1	***		*		*		*		*
**C5**	-0.615	-0.694	0.745	0.623	-0.884	-0.860	-0.770	0.999	1		*		*				
**C6**	-0.993	-0.974	0.955	0.991	-0.855	-0.880	-0.943	0.557	0.514	1	***	**	***	***	***	***	***
**C7**	-1.000	-0.994	0.984	1.000	-0.911	-0.931	-0.976	0.653	0.613	0.993	1	*	***	***	***	***	***
**C8**	0.648	0.565	-0.503	-0.640	0.279	0.326	0.469	0.152	0.202	-0.736	-0.650	1	*	***	**	**	**
**C9**	-1.000	-0.992	0.980	0.999	-0.902	-0.923	-0.971	0.637	0.596	0.995	1.000	-0.666	1	***	***	***	***
**C10**	0.861	0.802	-0.757	-0.855	0.576	0.616	0.731	-0.178	-0.128	-0.916	-0.862	0.946	-0.872	1	***	***	***
**C11**	-0.997	-0.984	0.968	0.996	-0.878	-0.901	-0.957	0.595	0.553	0.999	0.997	-0.704	0.999	-0.897	1	***	***
**C12**	-0.985	-0.961	0.938	0.983	-0.827	-0.853	-0.924	0.512	0.468	0.999	0.985	-0.771	0.988	-0.936	0.995	1	***
**C13**	-0.997	-0.982	0.966	0.996	-0.875	-0.898	-0.955	0.590	0.548	0.999	0.997	-0.709	0.998	-0.900	1.000	0.996	1

Correlation matrix of significant constituents of essential oil and quality indices in modulation ripening process under treatments. Here lower triangle represents correlation values and upper triangle represents the significance levels ('*', '**'and '***' represent significance level at $P \leq 0.05$, $P \leq 0.01$ and $P \leq 0.001$ respectively). HA= hue angle, FF= firmness of fruit, PWL= percentage of water loss, TSS= Total Soluble Solid Content, C1= Cuben-11-ol, C2= Spiroether, C3= 1,3-dihydroxypropan-2-ylhexadecanoate, C4= Methyl labdanolate, C5= Tetracosane, C6= Pentacosane, C7= Hexacosane, C8= Nonacosanoicacid, C9= Heptacosane, C10= Pentatriacontane-2-one, C11= Triacontane, C12= Hentriacontane, C13= Dotriacontane.

Table S2: Primer used in the PCR reaction

Gene Isoform	NCBI Accession Number of Gene	Forward primer	Reverse Primer	Product Size	References
**CaACO1**	GD053075.1	GCAAGTGCTTAAATTACAAG TGTG	TTGAGATGCAACCGTTACTCC	195	Aizat *et al.*, 2013 [22]
**CaACS1**	AB434926.1	AAGTTGTCGATGAAATGATT GG	CTAGCGATGTCCACTACTGTATCA	136	Aizat *et al.*, 2013 [22]
**CaETR1**	GD072810.1	AGAGTCCATGCGAGCCCA	CTTCTTGTCGAGCTACATCCAA	68	Aizat *et al.*, 2013 [22]
**CaETR2**	GD097854.1	TTGGGATGCAGCGTGTCT	AAGGACGATTTGGAATGAGG	87	*Aizat et al.*, 2013 [22]

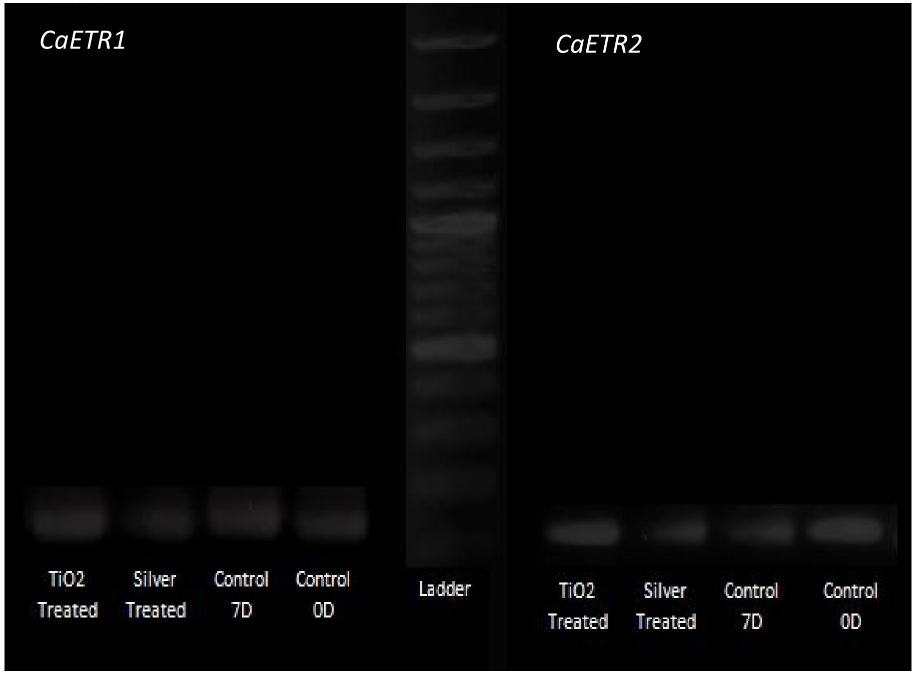

Figure S1: *CaERT1* and *CaETR2* gene expression bands under different treatments with 100bp DNA marker
